# Parent Training Occupational Therapy Program for Parents of Children with Autism in Korea

**DOI:** 10.1155/2017/4741634

**Published:** 2017-03-01

**Authors:** Sun-Joung L. An

**Affiliations:** ^1^Department of Occupational Therapy, Inje University, 197 Inje Rd, Gimhae-si, Gyeongsangnam-do 621-749, Republic of Korea; ^2^Department of Rehabilitation Science, Graduate School of Inje University, 197 Inje Rd, Gimhae-si, Gyeongsangnam-do 621-749, Republic of Korea; ^3^Ubiquitous Healthcare Research Center, Inje University, 197 Inje Rd, Gimhae-si, Gyeongsangnam-do 621-749, Republic of Korea

## Abstract

Attitudes and beliefs about parent participation in occupational therapy are shifting toward family-centered practice worldwide. However, adopting a family-centered approach in a society such as Korea, where a Confucian culture of hierarchical roles is reflected in a strong medical model, can prove to be very difficult. A parent training program was developed at the HOPE Center, a pediatric occupational therapy center, to bridge the gap between the traditional medical model and the ideal family-centered model. This study examined the effectiveness of the parent training and gauged parents' perceptions and experiences of a more family-centered approach to therapy. Four parent-child dyads living with autism participated in five months of parent training at the HOPE center. The results on the Canadian Occupational Performance Measure showed that the parent training improved the occupational performance of both children and parents. Six open-ended questions were used to investigate parents' perceptions and experiences of parent training. Two broad themes emerged: improved self-efficacy and the cultural reality of living with autism in Korea. This study demonstrates that building parent training into an occupational therapy program may optimize the effectiveness of any therapy and introduce a more family-centered approach to therapy while maintaining cultural integrity.

## 1. Introduction

In western society, there has been a shift in occupational therapy service delivery models for children from medical and child-centered models in the mid-1900s to home programs and then to a family-centered model in the 1970s [1–3]. The medical model focuses on a “health problem” and its treatment, where the health professional is viewed as the “expert” who brings treatment to the individual with the health problem. The roles of all participants in this medical model are clear: the health professional is the active and powerful agent of change and the individual with the health problem is the passive and dependent recipient of treatment. Family members have little to no role to play within this model except to facilitate engagement between the health professional and the individual patient. In contrast, the family-centered model recognizes that a health problem of an individual has a significant impact on the entire family and as such, any intervention must be guided by “the needs of the entire family—the parents, the siblings, and the child with special needs” [1]. Within this model, there is no single “expert” and all participants working together collaboratively optimize the benefits of any treatment. Whilst there has been a theoretical shift from the medical model to the family-centered model in western society, research shows that, internationally, therapists continue to struggle to work collaboratively with parents within a family-centered framework as it “requires a significant change in thinking from more traditional child-focused approaches” ([2] p. 14). It also requires active participation and acceptance of a new equality within a relationship that is not in line with traditional cultural roles. This is certainly true within the cultural context of Korea, where Confucian traditions continue to strongly influence the hierarchical roles reflected in the medical model of service delivery, including occupational therapy service delivery for children.

According to Kim-Rupnow [4], “service providers such as doctors, nurses, teachers and therapists are well respected in the Korean community and the consumers tend to listen to their advice and follow their directions as passive recipients” (p. 20). This is consistent with the Confucian philosophy that underpins the cultural tradition of Korea, a philosophy that emphasizes “harmony and order within a system of prescribed roles” ([4] p. 4). The “social relationships in Korean culture are based to a large degree on a hierarchy of differences that confer status” ([5] p. 516). Accordingly, whilst there is an increasing awareness about the benefits of family-centered practice, Korean pediatric occupational therapists continue to deliver services through a traditional medical model, focusing on the physical or mental impairments of a child. Cultural expectations require Korean therapists to work directly with the child only and do not extend to an inclusive, collaborative role of families [6].

Recognizing the overwhelming gap between the theory of the family-centered model and the cultural reality of the Korean occupational therapy landscape, the present author established the Health through Occupational Performance Enhancement (HOPE) Center at Inje University in South Korea. HOPE Center was not only established to be a pediatric occupational therapy treatment center but also established to be a* parent training center* in the deliberate attempt to gradually move towards a more family-centered model of therapy whilst maintaining culturally sensitive expectations. At the HOPE Center, a parent-child dyad is always present throughout all treatment sessions and parents are encouraged and* taught* to participate collaboratively with therapists in the treatment of the child.

Occupational Performance Coaching (OPC) is “a process whereby parents are guided in solving problems related to achieving self-identified goals” ([7] p. 16). The primary goal of OPC is “improvement in the performance and satisfaction families experience as they go about their everyday lives and the secondary intention is enhancement of parents' skills to resolve children's performance difficulties with greater autonomy in the future” ([7] p. 5). A critical concept within the OPC is* coaching*, where “therapists do not* tell* the parent what to do but rather therapists guide parents in developing strategies and supports to meet their family's needs” ([8] p. 253).

“Parent training” was introduced at the HOPE Center as a hybrid form of OPC, having the same primary goal of improving performance and satisfaction experienced by families, whilst modifying the concept of “coaching” to a more culturally acceptable form of* directing* parents, rather than* guiding* parents, to develop strategies and supports to meet the needs of families. Underpinning parent training at the HOPE Center is the fundamental principles of the family-centered model ([9] p. 79):

1. Parents know their children best and want the best for their children.
2. Families are unique and different.
3. Optimal child functioning occurs within a supportive family and community context.

This parent training at the HOPE Center comprises an introductory teaching component, where parents gain deeper understanding of occupational therapy and the roles of all participants in the therapy of the child. This teaching is reinforced throughout the child's therapy sessions, where the therapist first models intervention whilst providing explanations for the purpose behind any treatment and then stands back to allow family members to participate in the therapy. Through learning and active participation, the desired goal is for families to feel empowered and experience a more egalitarian model of service delivery.

After two years of operating, the aim of this research was to examine the effectiveness of the parent training program at the HOPE Center. Four cases of children with autism and the responsiveness of children and parents to a service delivery model involving parent training are presented, exploring two research questions:

1. Does this parent training approach to treatment improve the occupational performance of the child, the parent, or both the child and the parent?
2. What are the parents' perceptions and experiences of parent training?

## 2. Method

### 2.1. Participants

Parent-child dyads who attended the HOPE Center during the five-month period of the study and who met the inclusion criteria were recruited to participate in this study. These were three mothers and a paternal grandmother (all referred to as “parent” in this study for ease of reference) who all provided their consent and participated voluntarily in this study. Participant inclusion and exclusion criteria are as follows.

*Screening Criteria for Participants*

*Inclusion Criteria:*

Attended HOPE for at least 5 months and stopped attending all other therapies working on occupational performance skills
  Diagnosis of severe autism
  Reported to have severe behavioral problems
  Nonverbal

*Exclusion Criteria:*

  Attended HOPE for at least 5 months and continued attending other therapies working on occupational performance skills
  Diagnosis other than severe autism
  Does not have behavioral problems
  Able to use verbal communication

*Dyad 1: MC and His Paternal Grandmother*. MC is a 6-year-old boy who lives with his father and paternal grandparents during the week as his mother works in a different province. The paternal grandmother is the primary caregiver for MC and reported that MC did not respond to any verbal instructions and had tantrums when asked to participate in daily self-care activities such as putting on his socks and shoes. MC's grandmother performed those daily activities, including feeding him a very limited diet consisting of rice, beef, and water. MC reportedly refused to eat anything else and could not tolerate any food around his lips or hands or spilling on his clothes.

*Dyad 2: JB and His Mother*. JB is a 4-year-old boy who was reported to be hypersensitive to all textures and refused to be hugged or held by his mother. JB's mother found this particularly difficult as she felt that he was rejecting her. However, he was clearly rejecting all forms of tactile input and not just his mother, pushing everything away including food, clothes, and other people. This made feeding very difficult and JB's mother reported that she cried almost every meal because JB would refuse all food entering his mouth. Ultimately, JB's mother fed JB by restraining his hands and forcing food into his mouth. JB's mother also reported irregular sleep patterns: it was difficult for JB to fall asleep and when he did sleep, he would wake up regularly throughout the night and wander around.

*Dyad 3: GW and His Mother*. GW is a 9-year-old boy who was demonstrating oppositional behavior during daily routines such as leaving his home or entering his school. GW frequently engaged in stereotypic behavior, making unusual sounds and flapping his hands. He was nonresponsive when called by name, was unable to dress or feed himself, and did not participate in routine daily activities.

*Dyad 4: JY and Her Mother*. JY is a 3-year-old girl whose mother sought occupational therapy assistance as JY was very lethargic, did not interact with her surroundings (including her mother), and would cry when her mother made attempts to interact with her. JY cried incessantly, refused eye contact, and was nonresponsive when called by name. Although JY was able to walk independently, her parents and grandparents often carried her, even at home. The only activity in which JY seemed to actively engage and enjoy was eating. However, JY did not chew her food and swallowed large chunks of food. JY's mother sought assistance to engage JY in play activities.

### 2.2. Study Design, Data Collection, Measures, and Data Analysis

A mix of quantitative and qualitative data collection and analysis was used in this study. This was needed to obtain appropriate data for each research question.

The Canadian Occupational Performance Measure (COPM) is a criterion-based measure of occupational performance and satisfaction where participants indicate scores on a scale of 1 to 10, with higher scores signifying greater performance and satisfaction levels [10]. The COPM has been researched extensively and has been found to be a reliable, valid, and sensitive measure of goal-specific performance change [11]. A change in scores of 2 points or more between pre- and postintervention COPM scores indicates clinical significance [12].

To explore the first research question of improvements in occupational performance of the child, the parent, or both the child and the parent, the present author retrospectively reviewed the clinical records of all parent-child dyads who had participated in treatment at the HOPE Center for the five previous months and who otherwise met all the inclusion criteria in* “Screening Criteria for Participants.”* Four families were identified and recruited to participate in this study. As a matter of standard practice at the HOPE Center, the COPM is completed by parents at the commencement of treatment and after five months of treatment. Accordingly, this data was available from the clinical records for analysis for the purposes of this study.

The performance goals of the child and the performance goals of the parent using the COPM criteria can be seen as largely interdependent. For example, before intervention, MC would only eat rice and beef. This reflected not only the child's eating performance but also the parent's performance in presenting to MC a limited range of food. Therefore, “MC will eat and drink food other than rice, beef and water” is the performance goal of both the child and the parent.

Changes in the occupational performance of the child and parent and satisfaction levels based on the COPM are in Table 2. Due to the small sample size, statistical analysis was not undertaken.

Having identified the four families fitting the inclusion criteria and having obtained their consent to use their data for the purposes of this study, the parents were then asked at the conclusion of their five-month parent training to participate in a semistructured interview to explore the second research question regarding parents' perceptions and experiences of parent training. Adapting Foster et al. [8] open-ended questions with subsequent probes (such as “tell me more about that”), six open-ended questions were designed to obtain data regarding parents' perceptions of and experiences of the parent training program in terms of their understanding of the program and its impact upon them and their children. Each of the four parents was interviewed by the researcher at the end of the five-month parent training period in the researcher's office. The interviews took approximately 1 hour and each was voice recorded and transcribed verbatim.

In order to systematically and fully uncover the experiences of the parents, the transcript of the interviews was then provided to the parents to review and add further comment, if desired [13]. All four parents made further comments on their experiences at this stage of data collection which were recorded and added to the transcript. The present author then read and reread the revised transcript, using content analysis to identify themes and discrete chunks of data relating to the second research question. Coding was used to analyze parents' perceptions and experiences of parent training. The revised transcripts and coded analysis were submitted for comment to an independent therapist at the HOPE Center who had no prior involvement in the study but who had an appreciation of parent training. The independent therapist provided some comments but agreed with the researcher's codes and analysis. Finally the codes and themes were reviewed again with the supporting comments from the reviewer.

The interview questions are presented in Table 1.

Approval for this study was obtained from the Inje University Ethics and Review Board and all family members participating in this study provided informed consent.

### 2.3. Program Description

Upon commencement of the parent-child dyad in this study, the parent was asked to complete the COPM. Based on the responses of the parent and the needs of the child as identified by the parent, the researcher tailored a program for each individual parent-child dyad, identifying treatment goals for the child and training goals for the parent. As noted previously, these goals were interdependent, since they were based on co-occupations engaged in by parent and child. The treatment program for the child comprised occupational engagement and sensory integrative procedures. The dyads participated in at least 20 sessions, one hour each week for five months.

The training program for the parent comprised a combination of interactive learning sessions with hands-on training during the child's treatment. In the first month of the program, each parent attended four learning sessions of approximately two hours each, conducted by the researcher, where parents engaged interactively with content such as the following:

1. The question, what is occupational therapy?
2. The question, what is occupational performance?
3. Analysis of daily occupations
4. Family-centered practice and collaborating with health professionals
5. Sensory integration relating to children's occupational performance
6. Behavior management relating to occupational performance

The treatment program for the child was also the forum for parent training and over the 20 sessions of treatment for the child. There was a continuing dialogue between the therapist and the parent, identifying and analysing strengths, problem areas, and goals for both the child and the parent. For example, whilst observing JY's reluctance to participate in any play activities, the therapist encouraged the parent to identify the reasons why JY would not participate in play activities. In this way, a more meaningful performance goal could be developed collaboratively between the therapist and parent, not only for JY to participate in play, but also for JY's mother to have a positive expectation that JY would participate in play. The continuing dialogue was assisted by a process of implementation and feedback where video recordings of the child, the parent, the therapist, and their interactions were played back to the parent to engage in a process of observation, identification, analysis, and implementation.

## 3. Results

### 3.1. COPM Results

The COPM results demonstrated positive changes in both performance and satisfaction for each of the goals for the children and the parents, as illustrated in Table 2. Improvements in COPM scores were clinically significant, being a change of more than 2 points on all criteria. Improvements in the reported levels of satisfaction were generally greater than the improvements in the performance itself. Within these satisfaction levels, the results show a pattern of parents being more satisfied with the improvements in the occupational performance of the child than in their own improved performance.

*Note*. Change in scores of 2 or more points is considered clinically significant [12].

### 3.2. Interview Results

After analysis of the interview data, four major themes emerged about parents' perceptions and experiences, which are examined in more detail below:

1. Parents identified gaps in their understanding and gained new learning through the parent training.
2. Parents reported a shift in their own attitudes and expectations of their child.
3. Parents lamented the realities of living with autism in Korea.
4. Parents identified a need for more guidance and direction.

### 3.3. New Learning

All parents reported learning new information through parent training, including information about their own child and about child development in general, and learning to identify and support their child's occupational performance needs.

Parents reported that, through parent training, they realized how little they knew about their child, about their needs, and how to assist them. GW's mother reported that she “didn't think GW had a personality because all he did was make monstrous sounds and rock himself all day.” However, through parent training, she learned that GW had unmet sensory needs and when he was given appropriate levels of sensory input, he was able to interact with her appropriately.

Parents also reported learning specific strategies to use in interacting with their child including behavior management, sensory strategies, oral motor stimulation, and providing hand-over-hand assistance during occupational performance. MC's grandmother reported, “I cannot believe I have learned how to feed MC foods other than rice, beef and water! I feel so good that I can introduce new foods and feed him other things too….” Parents reported feeling “relieved and satisfied” when they had learned specific skills in facilitating their child's occupational performance.

Overlapping with the second emerging theme of parents' expectations, parents also reported gaining insight about themselves, their own perceptions about their child, and their interaction with their child based on their low expectations. JB's mother stated, “I just thought he couldn't do anything so I automatically did everything for him… I thought JB didn't look at me or respond to being called by name because of his diagnosis… Nobody told me that he could be facilitated to communicate in ways other than tantrumming.”

### 3.4. Shift in Parents' Attitudes and Expectations

A clear and encouraging theme emerged from this study of a reported shift in the parents' own attitudes towards their child. For example, JY's mother recognized that her attitude shifted from “my poor baby with a disability who can't do anything” to “my child who can be assisted to participate in daily activities.” Similarly, the parents of GW and JB also reported that the change in their own attitudes towards their child led to higher expectations of their child, which in turn positively influenced the child's occupational performance. GW's mother reported, “my husband's attitude and my attitude towards our son has changed since the learning sessions. We now expect GW to participate in daily activities much more… The funny thing is that with just that change in attitude, GW is doing so much more and is so much more responsive!”

Parents reported improved interactions with their child as a result of the shift in their attitudes and expectations. JB's mother stated, “I feel more comfortable holding JB and don't feel so rejected or hurt anymore when he pushes me away. I think that has reduced the tension between us… He has started to come and hug me – I never thought this would be possible! Remember all the scratches on my arms? I can cuddle my baby just like other children now.”

With the shift in attitudes and expectations, parents reported feeling less stressed interacting with their child. Instead they felt empowered to find ways to facilitate their child's occupational performance.

### 3.5. Living with Autism in Korea

“Autism is still not accepted in our culture, you know. We don't use the word ‘autism'. People prefer to use the term ‘borderline children' instead of autism,” says JY's mother. Another recurrent theme emerging from this study was the difficult reality of living with autism in Korea, the stigmatization, lack of support, feelings of isolation, and feeling bound all day to their extremely dependent child with autism. JB's mother reported that no other family members or friends were aware of JB's diagnosis of autism except JB's grandmothers; not even the grandfathers were made aware of the diagnosis.

All the parents described in detail how they avoided all family and social functions since they received the diagnosis of autism in their child. All parents expressed feeling isolated and feeling unable to be out in public with their child, not even to restaurants, for fear of being stigmatized and stared at. The parents of JB and GW reported their persistent attempts to find a therapist all over Korea who might “cure” their child so that their child would not have to live with the stigma of their diagnosis for the rest of their lives.

It became apparent that the strong familial connections and support that are available to most families in Korea are not available to the families participating in the study. For example, JB's mother reported that because JB's grandfather is unaware of JB's diagnosis, JB's grandmother refuses to care for JB when her husband is home. GW's mother reported moving away from her friends and family of origin to another city since GW's diagnosis because “my husband and I did not want others to know about GW's diagnosis. So I don't have anyone to help me look after GW. I can't afford to get sick since I moved away from my family.”

All parents reported feeling overwhelmed and stressed. Their entire days revolve around the needs of the child with autism, taking them to various therapies or taking care of their daily activities, with no support.

### 3.6. Desire for Further Direction and Guidance

Despite the emphasis placed on family-centered practice throughout the parent training program and despite the pleasing gains made in the occupational performance of both the child and the parent, parents continued in their desire for the therapist to be the authority to set goals and provide specific home programs. In response to the fourth interview question, “What do you want us to do differently in parent training?,” all parents answered that they wanted more direction, guidance, and goals set for them. Parents reported feeling incompetent and unqualified in setting goals for their own child and preferred to defer to the “expert” or “qualified professional” to set goals for their child and design specific home programs for them to simply follow.

Through parent training, parents reported a growing realization of their role as collaborating partners and the need for them to be more active participants in the treatment of their child with autism. However, they each defaulted to feeling more comfortable in being told what to do by the “expert professional.”

## 4. Discussion

The quantitative data in this study was reinforced by the qualitative data; the clinically significant improvements in the performance of both the child and the parent and satisfaction levels shown by the COPM results were supported by the overall reports of gains in self-efficacy through the parent interviews. The parents' experiences of parent training were generally positive, with parents reporting new learning and experiences that effected a shift in attitudes toward their children, which in turn effected improvements in the performance of parents and children. This is consistent with the findings of Graham et al. that “learning was a predominant feature of mothers' experiences of OPC, which may provide insights into the mechanisms that underpinned the changes observed in mothers' and children's performance” (2014, p. 194) and that parents gain “greater insight into the impact of their own emotional state and actions on their children's performance” ([11] p. 11).

This study demonstrated that, as parents' grew in their learning and understanding about child development in general and specifically about their own child's needs, their sense of feeling overwhelmed and stressed in their interactions with their child declined significantly and they felt “empowered to figure out ways they can improve the quality of their life and their kids” ([8] p. 260). Feelings of relief and satisfaction were reported by all participants as they tried new strategies with their child, demonstrating improved self-efficacy and echoing Graham et al.'s (2013) view that learning processes may explain links between learning and other intrapersonal changes such as improved self-efficacy.

The four themes emerging from the qualitative data can be further summarized in two broad themes: improved self-efficacy through new learning and shifting attitudes and beliefs and the cultural reality of living with autism in Korea determining the level of support that a family expects to receive from their community. The former develops a sense of intrinsic empowerment, and the latter promotes an extrinsic sense of powerlessness.

Hence, despite improvements in performance and satisfaction levels, parents reported difficulties of living with autism in Korea. “Autism means more than an impairment of the child” ([14] p. 545) and to the participants of this study, it meant feelings of unacceptance, isolation, stigmatization, lack of social support, and being bound by the burden of care for their child with autism. According to DeGrace, “a family's identity forms as the unit negotiates tasks of daily living and develops routinized ways of living” ([14] p. 548). The families in this study reported not being able to attend family functions, eat at restaurants, or go out in public. In negotiating their daily experiences within their family systems and wider social systems in these ways, the identity of their family units simply merged under the label of “autism,” as for the families in DeGrace's 2004 study.

Already living with autism in a defensive mode because of the cultural environment of nonacceptance and stigmatization, these families then demonstrated the depths of their cultural expectations to defer to their hierarchical role of passively receiving treatment and direction from the “qualified professional.” Participants did not see themselves as relevant “experts” and felt unqualified to be collaborating partners. The participants' request for further direction and guidance reflects the traditional view in Korea that health professionals are the authority in a medical setting [5]. With the prevalent medical model and emphasis on hierarchy in social relationships, both influenced by deeply rooted Confucian philosophy in Korea, this study demonstrates the difficulty in moving away from the medical model towards a more family-centered model of service delivery.

Overall, however, the results of this study are hopeful. The study was set to examine two research questions:

1. Does this parent training approach to treatment improve the occupational performance of the child, the parent, or both the child and the parent?
2. What are the parents' perceptions and experiences of parent training?

Whilst the study could not control for extrinsic factors such as cultural influences and expectations, it could affect intrinsic factors such as new learning, awareness, and improvements in self-efficacy. A clear improvement in occupational performance and satisfaction was demonstrated in this study through parent training over a five-month period. Five months of parent training cannot usurp hierarchical roles and expectations developed over centuries, but it signifies the opportunities for a movement towards a more family-centered practice based on promoting collaborative parent-therapist partnerships, with “service providers as technical experts with knowledge and perspectives on the condition and treatments and parents as experts on their child, their family, and their strengths, needs and values” [2].

## 5. Limitations and Future Directions

The participants were a homogenous sample of families of children with autism attending the HOPE Center for occupational therapy treatment and parent training program. Accordingly, the results do not represent the effects of parent training in families where children have other or no diagnoses. Future studies including participants with other or no diagnoses and comparing results with the present study would strengthen the validity of this study.

Participants in this study are also likely to be biased towards families with a predisposition in favour of parent training, as families who attended the HOPE Center were self-referred clients. The author's prolonged engagement and relationship with the participating parents and children may have influenced the interpretation of data. Future studies examining the effectiveness of parent training should consider the use of an interviewer who has not had a therapeutic relationship with the participants.

## 6. Conclusion

In an effort to begin to bridge the gap between the existing medical model in Korean pediatric occupational therapy settings and the ideal of family-centered practice, parent training was introduced at the HOPE Center. Parent training is a service delivery model that adopts culturally appropriate aspects of family-centered practice by including parents in therapy whilst recognizing the deeply held hierarchical roles within Korean culture and thus using the therapist's authority to train the parents.

The effectiveness of this parent training model was sought to be examined by posing two research questions:

1. Does this parent training approach to treatment improve the occupational performance of the child, the parent, or both the child and the parent?
2. What are the parents' perceptions and experiences of parent training?

The results from this study showed that parent training for mothers of children with autism was effective in improving the children's and mothers' occupational performance. It also showed improved self-efficacy through new learning and shift in attitudes and beliefs, that is, the capacity of parent training to develop an intrinsic sense of empowerment. At the same time and, unsurprisingly, the study reinforced the known cultural realities of Korea, leaving families of children with autism feeling powerless due to extrinsic factors: being unable to receive social support from family and friends and expecting to receive help from the expert professionals.

However, if five months of parent training can demonstrate clinically significant improvements in performance and satisfaction and can demonstrate improved sense of empowerment and self-efficacy arising from new learning and a shift in expectations, there is great hope that these positive movements can reduce the impact of stigmatization and increase the willingness of families to work more collaboratively in partnership with therapists. Ultimately, the goal of any therapy is not to conform to any particular model, but to optimize effectiveness and gains on all participants. This study has demonstrated that parent training empowers families within a culturally sensitive framework and has the potential to optimize the effectiveness of therapy. Ongoing examination of this parent training model in a variety of contexts would improve the practice, perceptions, experiences, and effectiveness of occupational therapy practice in Korea.

## Figures and Tables

**Table 1:** Open-ended Interview Questions.

Number	Question	Type
(1)	What do you think parent training is about?	Understanding
(2)	What do you think we were trying to do in parent training?	Understanding
(3)	What would you like to tell us about your parent training experience?	Impact
(4)	What do you want us to do differently in parent training?	Impact
(5)	What would you tell other parents about the parent training?	Understanding
(6)	What are you doing differently since you have attended parent training?	Impact

**Table 2:** COPM Scores for Parent Identified Goals for Themselves and Their Children.

Client	Goal	Performance			Satisfaction		
		Pre	Post	Change	Pre	Post	Change
MC	MC will eat and drink food other than rice, beef and water	2	5	3	2	7	5
	MC will turn and look when his name is called	2	6	4	3	8	5
MC's parent	Grandma will call MC by his name and look at him instead of just doing things for him automatically without talking to MC	2	6	4	3	7	4
JB	JB will interact with his mother and tolerate being held and hugged by her and also reciprocate hugs	1	7	6	1	8	7
	JB will tolerate being fed puree food without tantrums or biting his mom	1	4	3	1	6	5
JB's parent	JB's mom feels competent when feeding JB	1	6	5	1	6	5
GW	GW will put on, take off his shirt, pants, shoes and socks without crying or tantrums	1	6	5	2	8	6
	GW will respond and come when called	1	6	5	2	7	5
GW's parent	GW's mom will facilitate and guide GW to participate in self-care activities instead of automatically doing it for him	1	6	6	2	6	4
JY	JY will get on the swing by herself and be pushed	2	8	6	2	9	7
	JY will interact with her mom by hugging her and coming to her when she is called	1	6	5	2	8	6
JY's parent	JY's mom will give calm instructions to JY and will talk to her	2	6	4	3	7	4
